# Grandparents and Parents: An Essential Partnership for Longevity Societies

**DOI:** 10.1093/geroni/igab046.2830

**Published:** 2021-12-17

**Authors:** Paris Strom

**Affiliations:** Auburn University, Auburn University/Auburn, Alabama, United States

## Abstract

This presentation hypothesizes that an innovative collaboration by the adult generations will be necessary to enable conditions needed for family success in a longevity society. Unprecedented challenges of parents and grandparents are examined. Reasons why adults have to regard youth as a source of learning about their unique experiences in an age-separated society are explained. International curriculum development studies to support families of children from birth through adolescence are described. A curriculum that provides a common knowledge base about child and adolescent guidance is proposed to harmonize efforts of adults to support younger relatives. Curriculum for retirees should focus on continuing responsibilities other generations expect of them, learning about the lives of younger family members, and gaining awareness of parenting practices to reinforce lessons. Training volunteers in assisted living and long-term care facilities to be indigenous leaders of grandparent classes is discussed as a practical way to offer relevant learning and improve social support. Instruments are examined that assess ethnic relationships between adult generations, adults and adolescents, and track results of education intervention.

